# Molecular analysis of mutations for the adenomatous polyposis coli (APC) gene in Romanian patients with colorectal cancer

**Published:** 2008-11-15

**Authors:** M Toma, D Cimponeriu, A Pompilia, M Stavarachi, L Beluşică, I Radu, L Gavrilă

**Affiliations:** * Departamentul de Genetică Umană, Institutul de Genetică, Universitatea din Bucureşti;; ** Spitalul Clinic I. Cantacuzino, Bucureşti

## Abstract

Mutations in adenomatous polyposis coli (APC) gene have not been previously characterized among Romanian patients with colorectal cancer (CRC). We initiate this study to detect the mutations in APC gene in blood and tumor samples collected from 16 patients (10 men and 6 women) and blood samples from 21 first and second degree relatives of the patients. For this the presence of mutations in exons 6, 7, 12, 13, 14 as well as in regions B, L and W of exon 15 was investigated using PCR multiplex. In the same time, we have searched for 5 bp deletions at codon 1061 of APC gene by PAGE and SSCP methods. These methods allowed us to evidence identification of the presence of mutations in samples from 7 individuals. In one patient, was detected a deletion of exon 13th of APC gene both in DNA extracted from blood and tumor samples. Multiple deletions (e.g. in exon 6, 12, and in 15L and 15W regions) in DNA extracted from the tumor sample were detected, but not in DNA probe obtained from blood cells. We can speculate that these mutations are an example of genomic instability accompanying the malignancy. Till now, no mutation affecting 1061 codon of APC gene was identified in the patients investigated in our study.

## Introduction

The incidence of colorectal cancer in Romania was 17,74/100.000 inhabitants in the year 2000. This value significantly increases in the last years, colorectal cancer becoming one of the most common type of cancer and the second cause of death in humans. Almost 60% of Romanian patients with colorectal neoplasms have at presentation a metastatic disease. These figures sustain the necessity to introduce of genetic tests for identification of persons predisposed to the neoplasmic disease in the Romanian population [**1**, **2**].

The mutations in adenomatous polyposis coli (APC) gene [**3**] are directly involved in the development of human CRC [**4**, **5**, **6**, **7**, **8**]. Although several hundred of mutations have been identified in APC gene [**9**, **10**] (**Table 1**), the majority (68%) are frameshift mutations that result in truncated proteins with abnormal functions. 

**Table 1:** Distribution of germline and somatic mutation of the APC gene (modified after Christophe B and Thierry S, 1996)

	Frameshifts		Point mutation		Total
	*deletion*	*insertion*	*Missense*	*Nonsense*	
Germline	221(66%)	21(6%)	6(2%)	87(26%)	335
Somatic	209(52%)	52(13%)	12(3%)	129(32%)	402
Total	430(58,3%)	73(10%)	18(2,4%)	216(29,3%)	737

Germline mutations in this gene have been identified in the majority of the autosomal dominant inherited familial adenomatous polyposis (FAP) [**11**], while somatic mutations occurs in approximately 80% of sporadic cases.

The most common germline mutations occur in 1061 and 1309 codons of the APC gene. Their cumulated frequencies for these represent a third of all germline mutations identified in Caucasian population [**12**, **13**]. Over 60% of all somatic mutations in APC occur within <10% of the coding sequence. This region is delimited by codons 1286 and 1513 and is known as the “*mutator cluster region*” (MCR) [**7**, **12**, **13**].

APC function has been linked to the Wnt signal transduction pathway. In normal condition, APC contributes to degradation of α-catenin [**10**, **12**, **14**] and to cellular adhesion, migration and apoptosis [**13**, **15**, **16**]. Since APC inactivation is sufficient to initiate tumour development, APC mutation affect degradation of α-catenin and truncated APC fragments stimulate colorectal cancer cell migration and generate chromosomal instability.

This study was conducted to detect the mutations in APC gene in blood and tumor samples collected from 16 patients (10 men and 6 women) and blood samples from 21 first and second degree relatives of the patients.

## Materials and methods

Subjects and selection criteria

Blood samples collected from patients (10 men and 6 women) with CRC and from first and second degree relatives without clinical signs of CRC (9 men and 12 women) have been analyzed. Tumoral tissue prelevated during surgical resection of colon have been collected. These tumors have been localized in ascending colon [**1**], transverse colon [**3**], descending colon [**4**], sigmoid colon [**2**] and in rectum [**6**].

Disease history, including detailed information of potentially factors involved on the onset and evolution of CRC, and medical treatment were collected for each subject by a medical specialized team. All subjects gave their consent before inclusion in study. This research was approved by the hospital ethics review committee.

DNA extraction methods

Genomic DNA was extracted from all samples with a commercial DNA Purification Kit (Promega), according to the manufacturer’s instructions. Each sample was tested for amplification success using three different sets of primers and PCR products have been electrophoresed in an agarose gel.

Multiplex PCR for APC gene

**Fig. 1:**
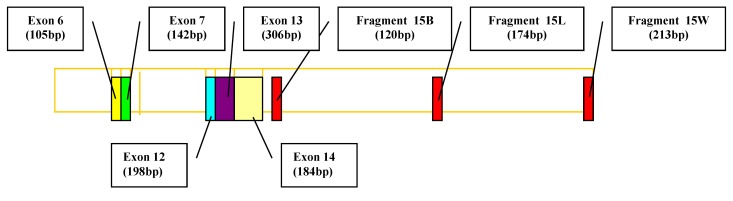
Schematic representation of APC gene and the relative position of analyzed segments are indicated by arrows. The sizes of amplicons are presented in bold type.

For this study, the initial protocol [**17**, **18**] was slightly modified for identification of mutations in 7th region of APC gene (i.e. exons 6, 7, 12, 14, and fragments 15B, 15L and 15W of exon 15) by a multiplex touch-down PCR and one region within exon 13 of APC gene in a separate PCR reaction. A fragment of 150 bp within exon 11 of BRCA1 gene has been used as an internal control in all PCR reactions (**Fig. 1**).

For a total volume of 10 μl, the mix contained: 100ng DNA, 0,25μl dNTP, 5μl PCR buffer, 0,1μl of each primer, and 0,3μl Taq DNA polymerase. The PCR conditions were: 3 min denaturation, at 95°C; 30 cycles at 94°C for 45 sec, 50°C, for 45 sec and 72°C, for 45 sec; final extension was at 72°C, for 3 min. Amplicons were resolved by electrophoresis in neutral polyacrylamide gel (8%).

Direct testing for 5 bp deletion starting at codon 1061

The region in which a deletion of 5bp (ACAAA) starting at codon 1061 has been reported, was amplified with a specific pair of primers (17). PCR products were obtained using the following protocol: 1 min denaturation at 94°C; 30 cycles at 94°C for 45 sec, 55°C, for 30 sec and 72°C, for 30 sec; final extension was at 72°C, for 2 min. Amplicons were resolved on PAGE (12%) and were visualized after ethidium bromide staining.

*Single-strand conformation polymorphism (SSCP) conditions.*

Two microliters of amplicon were denatured by mixing with 8 microliters of a stop solution (95% formaldehyde, 0.05% bromphenol blue and 0.05% xylene cyanol), heated at 95°C for 5 min, and quenched on ice. Amplicons were resolved by electrophoresis in neutral polyacrylamide gel (12%) and the gels were visualized after silver staining.

## Results and Discussions

There is no information regarding the type of mutations in APC gene or their frequency for Romanian population. The study was conducted over a period of 12 months. The characteristics of the subjects included in study are summarized in **Table 2**.

**Table 2:** Summary description of patients and relatives lots.

Characteristics	Patients	Relatives
No	16	21
Gender (M/F)	10 /6	9/12
Mean age +/- SD at diagnosis (yr)	70+/-9,41	51+/-8,5
Clinical signs	Various stages	Absent
Risk factors	Colonic diverticulosis(1), gastric polyp(1), diabetes(2)	

**Fig. 2:**
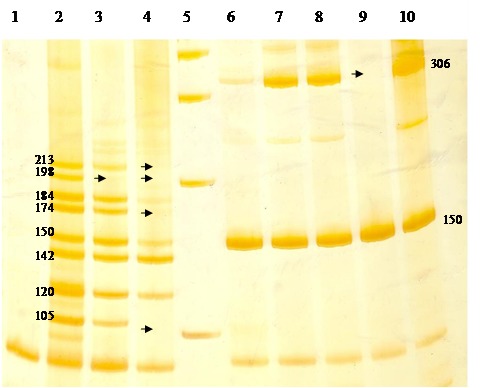
Silver-stained polyacrylamide gel of APC PCR Multiplex (left) and PCR for exon 13 (right). Line 2-4 - the results of PCR multiplex for exon 6, 7, 12, 14, and fragments 15B, 15L and 15W; Line 6-10 – the amplicons for exon 13 of APC gene. Line 1 - negative control; Line 5 – 100bp DNA ladder; The deletion is indicated by “->”.The 150 bp amplicon corresponds to internal PCR control (e.g. exon 11 of BRCA gene). The sizes of amplicons are presented at the right side of line 2 and at the left side of line 10.

In one patient, the deletions of exons 6, 12 and of fragments 15L and 15W of exon 15 in DNA extracted from the core of the tumoral tissue were detected. These mutations have not been detected in DNA extracted from blood samples. This can lead to the speculation that these mutations can represent an extensive deletion triggering the genomic instability observed in tumoral tissue. In other patient, a deletion of exon 13 in DNA extracted both from blood and tumor samples was detected (**Fig. 2**).

**Fig. 3:**
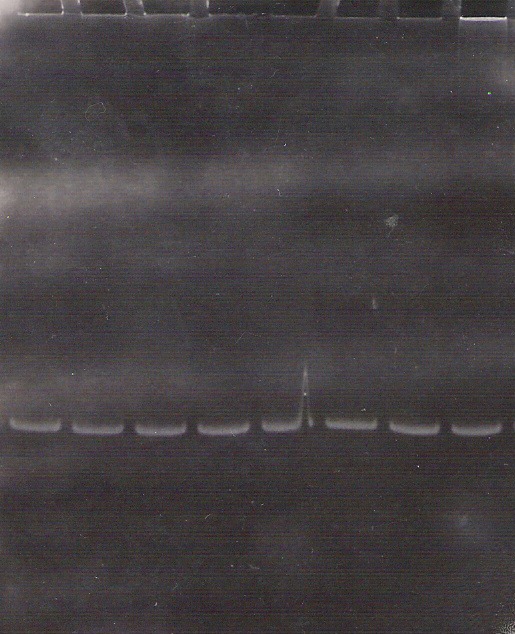
Electrophoresis (PAGE 12%) of amplicons for testing region around codon 1061.

In our study, the presence of 5 bp deletion starting in codon 1061 of APC gene was tested. No deletion in this region in patients as well as in their relatives was found (**Fig. 3**). 

**Fig. 4:**
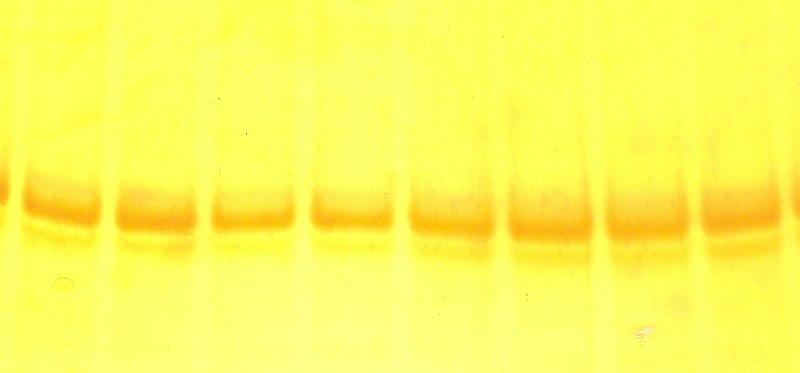
Silver-stained polyacrylamide gel of PCR SSCP fragment containing the codon 1061 of APC gene.

In order to detect unknown mutations within the region including codon 1061, the SSCP analysis was employed, since this method has higher sensitivity in the detection of point mutations. Because similar electrophoretic running pattern was observed for all samples, we consider that in this region no mutations were produced (**Fig. 4**).

The examined samples were constituted from 16 patients (10 men and 6 women) with CRC and 21 first and second degree relatives without clinical signs of CRC for occurrence of somatic and germinal mutations in the APC gene. 

We start our research by screened a part of the APC gene on the basis of previous studies [**17**, **18**, **19**, **20**].

The frequencies and the specific types of mutations in APC gene were investigated. In a number of studies, somatic mutations are reported to be present in 34 to 70% of sporadic CRC. The majority of mutations that determine a truncated and an inactivated APC protein are nonsense point mutations and frameshift mutations [**9**]. Depending on the phenotype of patients examined and the methods used for mutation analysis, germline mutations have been detected in 30-85% of FAP families. 

For example, in a study based on tumour tissue from 665 colorectal cancer patients: 72% of tumours have exhibited 978 mutations in the MCR of APC gene; 1 to 8 mutations per tumour were observed; the frameshift mutations seem to cluster in the regions delimited by codons 1350-1356, 1411-1419, 1465, 1485-1495 and in codon 1309; a high frequency of nonsense point mutations was detected at codons 1294, 1306, 1328, 1367, 1378, 1406, 1429 and 1450; a large number of missense mutations were distributed rather evenly throughout the MCR of APC and did not exhibit distinct hot spots [**19**].

The frequency of germline mutations varies depending on populations. For 5bp deletions starting in codons 1309 and 1061 the frequencies reported were as following: in Australian populations 2,4%, respectively 8,4% [**21**]; in Dutch populations 6,9%, respectively 4,9% [**22**]; in Israeli population 7%, respectively 1,5% [**23**]; in Polish population 15,2%, respectively 5% [**244**]. No mutation for codon 1309 was identified in NortWest Spain population [**25**].

Our study is based on molecular genetics approach and was performed in a relative small lot (n=37). However, it was enough relevant to reveal mutations in eight region of APC gene that were tested by specific methods. This represents the initiation of implementation of new approaches searching for APC mutations detection in patients with CRC in Romanian population. At the same time, it assesses the possibility of using molecular analyses to improve populational epidemiologic screening in order to offer a real and productive genetic counseling and diagnosis for those persons being at risk for CRC.

In present, by developing our project we looking to increase the size of studied lots and optimizing new testing protocols for mutations in APC gene in order to enhance the sensitivity of the detection and to have possibility to apply statistical methods in order to avoid false positive results. The low penetrance variants of the genes that can contribute to the risk for colorectal cancer in Romanian population must also to be taken into consideration. 

**Acknowledgments:** This research was supported by the CNCSIS (project A, 900)
